# Dataset’s chemical diversity limits the generalizability of machine learning predictions

**DOI:** 10.1186/s13321-019-0391-2

**Published:** 2019-11-12

**Authors:** Marta Glavatskikh, Jules Leguy, Gilles Hunault, Thomas Cauchy, Benoit Da Mota

**Affiliations:** 1 LERIA, University of Angers, 2 Bd Lavoisier, 49045 Angers, France; 2 Laboratoire MOLTECH-Anjou, UMR CNRS 6200, SFR MATRIX, UNIV Angers, 2 Bd Lavoisier, 49045 Angers, France; 3 HIFIH, EA 3859, Institut de Biologie en Santé PBH-IRIS, CHU, University of Angers, 4, Rue Larrey, 49933 Angers, France

**Keywords:** Molecular chemistry, SchNet, QM9, PC9, DFT

## Abstract

The QM9 dataset has become the golden standard for Machine Learning (ML) predictions of various chemical properties. QM9 is based on the GDB, which is a combinatorial exploration of the chemical space. ML molecular predictions have been recently published with an accuracy on par with Density Functional Theory calculations. Such ML models need to be tested and generalized on real data. PC9, a new QM9 equivalent dataset (only H, C, N, O and F and up to 9 “heavy” atoms) of the PubChemQC project is presented in this article. A statistical study of bonding distances and chemical functions shows that this new dataset encompasses more chemical diversity. Kernel Ridge Regression, Elastic Net and the Neural Network model provided by SchNet have been used on both datasets. The overall accuracy in energy prediction is higher for the QM9 subset. However, a model trained on PC9 shows a stronger ability to predict energies of the other dataset. 
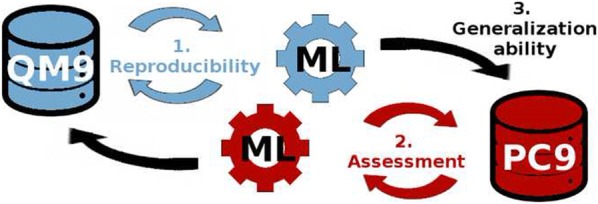

## Introduction

Quantum mechanical (QM) calculation is so far the most accurate method to obtain molecular energetic characteristics like the total energy and the frontier molecular orbitals energies (HOMO and LUMO). However, a huge computational cost prevents its daily usage for exhaustive exploration of chemical space. Till recently the only alternative in overcoming the time factor was to use less accurate approximation of QM or classical molecular mechanics. Yet, with those methods, the gain in time means a loss in precision. An appealing alternative is to use a computationally much more efficient approach based on machine learning (ML) models, which could be trained on any kind of data. It is indeed a hot topic. Just in 2019, an impressive amount of studies have been devoted to the application of ML for the prediction of molecular energetic characteristics [1–11].

In chemistry-oriented ML, especially for molecular energies, the employed methods encompass Kernel Ridge Regression (KRR) [12–18], sometimes Gaussian process regression (GPR), linear regressions (Elastic Net, Bayesian Ridge Regression) and Random Forest (RF) [17, 19, 20]. The most recent state-of-the-art predictions have been obtained by neural networks (NN) [1, 2, 21–28]. ML performance as well depends on the molecular representation used. Graph representations can be employed [23, 25], but the most common approaches are based on derivatives of geometry features like the Coulomb Matrix (CM) proposed by Rupp et al. [12], the Bag of Bonds (BoB) proposed by Hansen et al. [13] or Bonds and Angles ML (BAML) descriptors [16, 18]. More complex representations have been also proposed. The idea behind those “alchemical” descriptors is to introduce a part of the Hamiltonian before the ML treatment for better predictive power and generalizability. We can cite for example smooth overlap of atomic potentials (SOAP) paired sometimes with Gaussian approximation potential (GAP) [19, 29, 30] and multidimensional distributions of interatomic many-body expansion [31].

Furthermore, the ML performance depends heavily on the dataset size and quality. Most of the works related to ML modeling of quantum chemical properties are using either the QM7(b) collection or its enlarged version QM9 [21, 32]. They are both subsets of the combinatorially enumerated chemical universe GDB up to 13 or 17 heavy atoms [33]. Whereas the QM7 is composed of 7211 molecules (C, H, N, O, S and Cl), the QM9 dataset encompasses 133,885 molecules with up to nine “heavy” atoms from the range C, O, N and F. The geometric, energetic, electronic, and thermodynamic properties of theirs were computed with a DFT method at the B3LYP/6-31G(2df,p) level of theory. This dataset has become a classical benchmark for ML studies due to its homogeneity, purity and lack of noise.

So, since 2012, the ML modeling of total molecular energies and the HOMO/LUMO energies, among other properties, has been the focus of several studies. Table 1 presents the evolution of the published mean absolute errors (MAE) reported in the literature. Clearly, with QM7 and QM9 datasets, a well tuned KRR model can almost reach chemical accuracy for the three properties with descriptors like the BoB or the BAML [16, 17]. With more complex “alchemical” representations and radially scaled KRR /GPR, they can even compete with the best Neural Networks results [30]. Such accuracy is really tempting, taking into account the relatively low computational cost of KRR/GPR methods.

**Table 1:** Mean absolute errors for atomisation energies $U_0$ in kcal/mol, HOMO and LUMO energies (in eV) for several models Kernel Ridge regression (KRR), Elastic Net (EN), Gaussian process regression (KRR), and neural networks (NN) reported in the literature (from oldest to most recent)

References	ML method/descriptor	Dataset	(Training–Test sizes)	$U_0$	HOMO	LUMO
Rupp [12]	KRR/CM	QM7	(7000–165)	10.0	–	–
Montavon [21]	multitask NN	QM7b	(CV 5000–2211)	3.7	0.15	0.13
Hansen [14]	KRR/BoB	QM7	(CV 5732–1433)	1.5	–	–
Huang [16]	KRR/BoB	QM7b	(5011–2200)	1.8	0.15	0.16
Huang [16]	KRR/BAML	QM7b	(5011–2200)	1.2	0.10	0.11
Faber [17]	EN/CM	QM9	(CV 118k–13k)	21.0	0.34	0.63
Faber [17]	EN/BoB	QM9	(CV 118k–13k)	13.9	0.28	0.52
Faber [17]	KRR/CM	QM9	(CV 118k–13k)	3.0	0.13	0.18
Faber [17]	KRR/BoB	QM9	(CV 118k–13k)	1.5	0.09	0.12
Faber [17]	KRR/BAML	QM9	(CV 118k–13k)	1.2	0.09	0.12
Bartók [19]	GPR/SOAP-GAP	QM7b	(5411–1800)	0.40	–	–
Bartók [19]	GPR/SOAP-GAP	QM9	(100k–31k)	0.28	–	–
Gilmer [23]	NMP NN	QM9	(120k–10k)	0.45	0.04	0.04
Smith [22]	ANI-1 NN	ANI	(13.7M–1.7M)	<1.5	–	–
Hou [26]	multitask NN	QM9	(119k–13k)	44.0	0.38	0.63
Schütt [24]	SchNet NN	QM9	(CV 110k–10k)	0.32	0.04	0.03
Lubbers [27]	HIP-NN	QM9	(CV 110k–20k)	0.26	–	–
Unke [28]	HDNN	QM9	(CV 100k–30k)	0.41	–	–
Willatt [30]	KRR/SOAP	QM9	(CV 100k–30k)	0.14	–	–
Unke [2]	PhysNet NN	QM9	(CV 110k–20k)	0.14	–	–

CV denotes a cross validation procedure. Since NN descriptors can be quite complex, they have been omitted

Alongside with kernel methods, several Neural Networks (NN) have been proposed and tested for the prediction of energetic and electronic properties of QM9. Complex NN architectures have been carefully designed to achieve good predictive performances. For instance, some convolutional layers are inspired by the “alchemical” descriptors mentioned above. Such models can be expected to have a good capacity for generalization. The precise description of those NN architectures is beyond the scope of this article. Gilmer et al. have proposed a framework called Message Passing Neural Networks (MPNNs), that shares common attributes of several promising existing NN models for graph structured data and uses bond type features in addition to interatomic distances [23]. It achieved exciting performances on QM9 benchmark where 11 out of 13 properties were predicted within chemical accuracy (1 kcal/mol on total energies and 0.1 eV for orbital energies). It is worth noting here that the reported values in the literature correspond to scores on test sets (a random sample of the dataset unseen during training) generally after cross validation (average over different runs with different subsets of data). The next study was a neural network engine for molecular energies called ANI, representing transferable neural network potentials and utilizing a Behler and Parrinello symmetry functions to build single-atom atomic environment vector [22]. ANI has been shown to predict total molecular energies at the level of < 1.5 kcal/mol albeit with a much larger training set size (a subset of GDB-11). This model has been very recently refined and errors in total energies prediction equal to 0.14 kcal/mol were reported by Smith et al. [1] Schütt et al. have proposed a deep tensor neural network (DTNN) to mimic many-body Hamiltonians [34]. Then they have introduced continuous filter convolutional layers as novel building blocks for deep neural networks [24, 35]. The architecture has been called SchNet. The reported accuracy achieved by SchNet on QM9 is 0.32 kcal/mol for $U_0$ and 0.04 − 0.03 eV for HOMO and LUMO energies. Finally, Willatt et al. published a KRR model with a SOAP descriptor and Unke et al. a complex NN architecture PhysNet. Both also reach a MAE of 0.14 kcal/mol on total energies [2, 30]. The advances in accuracy achieved for energetic properties of QM9 is truly enthusiastic. However, the correspondence of the virtual combinatorial dataset to subsets of real molecules has not yet been examined. The expectation of a scientist working with a custom dataset, regarding the performance achievable by the variety of ML methods and tools built upon QM9, could hence be overestimated.

We propose here to address to the largest database of existing compounds, PubChem [36, 37]. Approximately $\approx$ 3 million molecules in PubChem have been calculated by Nakata et al. within the framework of PubChemQC project, with a DFT method at the B3LYP/6-31G(d) level [38]. In this article we have applied the limitations of QM9 dataset (size of up to 9 heavy atoms in the range C, N, O and F) to isolate a new dataset. Named PC9, it can be used for benchmarking comparison with QM9.

**Fig. 1:**
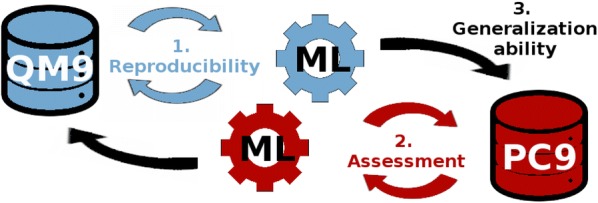
Workflow of this study

This study is constructed in accordance with the workflow given in Fig. 1. In the context of our research, we started by reproducing simple models, ie. Elastic Net (EN) and Kernel Ridge Regression (KRR) with Coulomb Matrices (CM), on QM9 and trying to assess their performances on PC9. As a first more complex model we began to look at SchNet, which was easily available. Results suggest generalization issues. So, in the last part we investigate the role of the dataset.

In the reproducibility and assessment parts, the comparison with QM9 results has been made possible by an identical modeling protocol. In the generalizability part, the ability to predict molecules of the other dataset, that were not seen during training, was evaluated. It consists in testing a model, trained on one of the datasets, for its prediction performance on molecules of the other dataset notwithstanding of their different chemical nature, occasional conformational discrepancies and different level of DFT theory. This topic is indeed almost never treated. Collins et al. has shown that a KRR model trained on QM7 and applied on QM9 displays an increase in MAE for $U_0$ from 3.4 to 106 kcal/mol with CM and from 2.4 to 30 kcal/mol with BoB [18]. However, HOMO and LUMO energies accuracy has been maintained around 0.15 eV. As it has already been observed by Faber et al. [31], the association of KRR and CM or BoB descriptors generalize badly compared to models which decompose the energies into atomic contributions. Can a complex NN architecture like SchNet that does such decomposition generalize better?

The last part of this article is devoted to the study of the differences between QM9 and PC9 in terms of chemical composition, abundance of functional groups and bond length distribution. Similarities and differences between the two datasets are exposed and visualized, providing insights concerning the ML performances.

## Method

### PubChemQC subset: PC9

Contrary to the pure in silico approach of QM9, PubChemQC represents so-far the largest quantum chemistry database of publicly reported molecules [38]. PubChemQC calculations are based on a DFT approach at the B3LYP/6-31G(d) level of theory and comprises more than 3 million molecules. The ground state geometries were optimized but no frequency calculations have been performed. To compare the ML predictions on QM9 and PubChemQC datasets, a congruent subset that meets QM9 limitations, i.e. restricted to up to 9 heavy atoms of the series H, C, N, O and F has been extracted. Chemical species were compared using the IUPAC International Chemical Identifier (InChI) notation [39]. The InChI for PubChemQC have been generated from the 3D Cartesian coordinates using the Open Babel software (version 2.4.1) [40]. A comparison has been performed between the InChI strings of the two datasets without the enantiomeric sublayer. Out of 3 million molecules in PubChemQC database, 118,662 met QM9 limitations. Out of this number, 99,234 InChI were unique since enantiomers, tautomers, isotopes or specific artefacts in PubChem lead to duplicated InChIs.

Different conformations for same chemical structure have been observed comparing QM9 and PC9. This point, as well as the composition and chemical function diversity of the two datasets, will be discussed further in section of generalization ability of SchNet models (see "Generalization ability of SchNet neural networks models" and Chemical differences between QM9 and PC9 sections). The prepared subset of 99,234 molecules is composed of two groups, the one comprising molecules that also belong to QM9 and the one with the molecules unique for PubChemQC. The former encompass 18,357 compounds and the latter contains 80,877 compounds. Hereafter we will only discuss the set of 99,234 molecules, which is indicated as PC9, but not the whole PubChemQC data.

As opposed to QM9, where all the molecules are constrained to be closed-shelled neutral compounds, a crucial point of PC9 is the presence of species whose multiplicity is $> 1$. That represents 5,325 molecules out of 99,234. Among which, 4442 are radicals ($m_s = 2$) and the remain 883 have a multiplicity of 3 (triplets). The prediction of those compounds has been analyzed separately and discussed further below.

### Predicted properties

Three key energetic properties have been considered in this article: total molecular energy E or $U_0$ (kcal/mol), energy of HOMO (eV) and energy of LUMO (eV). In QM9 dataset those values are readily available. However, the $U_0$ incorporates the zero point vibrational energy (zpve), when the total (SCF) energies of PubChemQC do not include it. Therefore, when speaking of reproducibility of the reported QM9 modeling results ( "Reproducibility of QM9" section) we refer to the original $U_0$ values, while the part of generalization ability of SchNet models ("Generalization ability of SchNet Neural Networks models" section) implies recalculation of E from $U_0$ in QM9 by subtraction of zpve, $E = U_0 - zpve$. Thus, both QM9 and PC9 datasets were attributed with a property of the same type.

It is important to mention that during modeling procedure, the atomization energies were used instead of total energies E (or $U_0$), for all ML methods. The atomization energy is the energy that remains after subtraction of the energies of all constituent atoms from the total energy of a molecule. Prediction of atomization energies instead of total energies is a common practice and is done in order to facilitate the training and to lower the magnitude of the property. The values of HOMO and LUMO were left in its original.

### Molecular representation

As stated in the introduction, several molecular representation have been used. The most common one is the Coulomb Matrix (CM) [12]. CM is a square atom by atom matrix constructed from atomic nuclear charges (Z) and Cartesian coordinates of each atom (R):

$$\begin{aligned} C_{II} =\, & {} 0.5 Z_I^{2.4} \\ C_{IJ} =\, & {} \frac{Z_I Z_J}{|\mathbf R _I - \mathbf R _J|} \quad I \ne J \end{aligned}$$

The off-diagonal elements of the CM then correspond to Coulomb nuclear repulsion terms whereas the diagonal elements approximate the electronic potential energies of the free atoms. Atom indexes in CM can then be sorted. This representation is invariant under translations and orthogonal transformations of Cartesian coordinates as well as under permutation of the indexing order of atomic numbers. Prior to modeling, CM have been standardized, i.e. scaled to zero mean and a standard deviation equal to one.

Neural Networks training has been performed with SchNet, whose predefined architecture implies row Cartesian coordinates as an input. The initial coordinates are then transformed by the algorithm in a set of layer-dependent features [35].

### Machine learning techniques

#### Kernel ridge regression (KRR)

KRR represents a non-parametric form of ridge regression (linear least squares with L2-norm regularization) combined with kernel trick. The goal is thus to find a linear relationship in a space induced by the corresponding kernel by minimizing a squared loss:

$$\begin{aligned} C = \frac{1}{2} \sum (y_i - w^T x_i )^2 + \frac{1}{2} \lambda \Vert w\Vert \end{aligned}$$

where *w* is the matrix of weights, *x* is the predicted value, *y* is the reference value. A closed solution form could be written as:

$$\begin{aligned} w = y (K +\lambda I )^{-1} \end{aligned}$$

where *K* is the kernel matrix. The parameters of the method were set up in accordance with Faber’s work [17]. The modeling has been performed with the scikit-learn package [41]. The regularization parameter $\alpha$ has been set to $10^{-9}$. Two types of kernels, Laplacian and RBF were used. Their widths have been chosen by a grid search on base-2 logarithmic grid (from 0.25 to 8192 for RBF kernel and from 0.1 to 16,384 for Laplacian kernel) for 10% of training set. Prior to learning, the feature vectors were normalized by the Euclidean (RBF kernel) or Manhattan (Laplacian kernel) norms.

#### Elastic net (EN)

EN [42] could be represented as a combination of lasso regression [43] and ridge regression [44]. It is a linear model with the penalty being a mix of L1 and L2 terms. Similar to modeling with KRR, EN hyperparameters were tuned up in a way it has been described in the article of Faber [17]. The l1 ratio was left at the default value of 0.5. A grid search has been performed to find an optimal regularization parameter ($\alpha$) on a base-10 logarithmic scale from $10^{-6}$ to 1.0 on the whole dataset. It is by far, the simplest and fastest method. It can serve as a crude approach to highlight the added value of more complex approaches, like NN.

#### SchNet neural networks

NN is a class of ML algorithms whose structure is modelled after the paradigm of biological nervous system functioning [45–48]. The architecture of NN consists of a network of neurons interconnected via weights and arranged in layers. The SchNet deep learning architecture is a variant of deep tensor neural networks (DTNN) and its architecture therefore includes such blocks as atom embeddings, interaction refinements and atom-wise energy contributions [24, 35]. At each layer, the atomistic system is represented atom-wise and refined using pairwise interactions with the surrounding atoms. To deal with unevenly spaced data such as atom positions, SchNet is provided with the continuous-filter convolutional layer (cfconv) that models the interaction term. The detailed architecture of SchNet is explained in Ref. [24].

In accordance to the original publications, the following parameters have been used while training: initial learning rate $10^{-4}$, batch size 32, number of features 256, number of interaction blocks 6, learning rate decay 0.5. The size of the training set has been set to 110,000 molecules, 1000 were used for early stopping and the remain quantity was assigned to test set.

## Results and discussion

### Reproducibility of QM9

#### EN and KRR

Following the original work [17] and with some guidance by Faber, we were able to reproduce the EN and KRR results on QM9 using Coulomb Matrix as molecular descriptors (see Tables 1 and 2). For $U_0$ a MAE of 4.9 kcal/mol has been obtained instead of 3.0 kcal/mol for KRR. Small changes have been made in the grid search to estimate the best hyperparameters. It has been done once in a 10-fold cross-validation on the whole dataset for EN and on 10% of a dataset (randomly chosen) for KRR. The best $\alpha$ for EN was always 0.001 and the best $\gamma$ of KRR were 1.0 ($U_0$), 2.0 (HOMO) and 4.0 (LUMO). Like for Faber, Laplacian kernel performed best in all three cases. The model performance has been evaluated during repeated random shuffling [20 times (EN)/5 times (KRR)] with 90% data being training set and the remain 10% being test. The average statistics is referred in Table 2. KRR requires a strict hyperparameter adjustment. Its performance is more sensitive toward data selection and may lead to different results depending on the training subset. EN and KRR can be trained to predict E with almost the same precision as for $U_0$.

**Table 2:** Mean absolute errors for total energies ($U_0$ and E in kcal/mol), HOMO and LUMO energies (in eV) using different ML methods on different training and prediction datasets

Method	Train	Test	$U_0$	E	HOMO	LUMO
EN (CM)	QM9	QM9	21.1	22.0	0.34	0.64
KRR (CM)	QM9	QM9	4.9	5.2	0.18	0.25
SchNet	QM9	QM9	0.3	1.0	0.04	0.03
EN (CM)	PC9	PC9	–	38.2	0.47	0.66
KRR (CM)	PC9	PC9	–	22.8	0.31	0.36
SchNet	PC9	PC9	–	1.6	0.06	0.05
SchNet	QM9	PC9(A)	–	3.0	0.07	0.06
SchNet	QM9	PC9(B)	–	8.9	0.33	0.27
SchNet	PC9	QM9(A)	–	3.4	0.05	0.05
SchNet	PC9	QM9(B)	–	4.2	0.12	0.11

(A) corresponds to the subset of molecules that belongs to QM9 and PC9, whereas (B) indicates molecules exclusive to the dataset

#### SchNet NN

The reproduction of QM9 predictions with SchNet is made easier owing to the recently published SchNetpack toolbox [49]. According to the reported results, the accuracy of SchNet that could be achieved with a training size of 110,000 compounds on QM9 is around 0.3 kcal/mol for $U_0$, 0.04 eV for HOMO energies and 0.03 eV for LUMO energies. The verification of this result could be done following the modeling procedure described in the source paper and thanks to indications from K.T.Schütt and M.Gastegger [35]. After $\approx$ 18 hours of calculation and passing from 300 to 500 epochs per property, the training reached MAE of 0.32 kcal/mol for $U_0$, 0.04 eV for HOMO and 0.03 eV for LUMO (see Table 2). It is worth noting that the performance of SchNet on QM9 using the total scf energies E correspond to an increased MAE of 1.0 kcal/mol keeping the same training parameters as for $U_0$, loosing more precision than the KRR model when going from $U_0$ to E. A factor of 3 in the MAE of the predictions for similar properties could indicate a generalization issue. Nevertheless, we confirm here that SchNetpack toolbox can easily and effectively predict within chemical accuracy molecular properties of QM9 dataset.

### PC9 modeling results

The same three ML algorithms were considered on PC9 dataset: EN and KRR trained on Coulomb Matrix and SchNet NN. The whole set of 99,234 compounds has been used. Grid search for best parameters led to $\gamma$ 2.0, 8.0 and 8.0 (E/HOMO/LUMO) for KRR (Laplacian kernel) and $\alpha$ value of 0.001 (E/HOMO/LUMO) for EN.

**Fig. 2:**
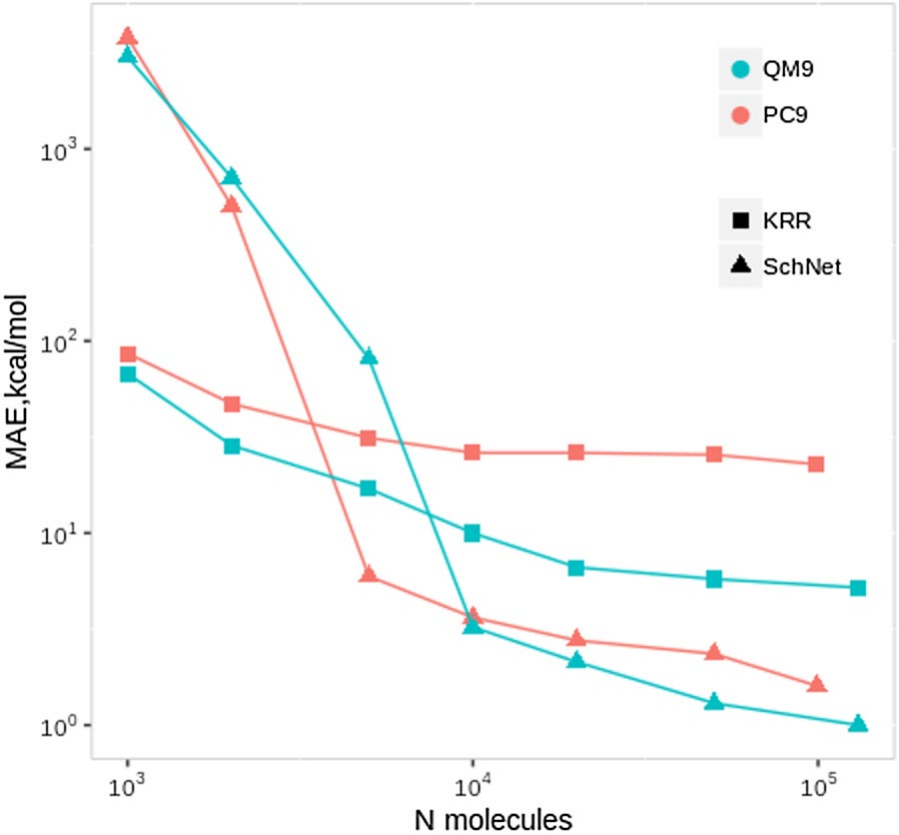
Mean absolute error assessed by a 10 fold cross validation with an increasing training set size for KRR and SchNet models. Training and test sets belongs to the same data set, either QM9 or PC9

The results of the modeling are given in Table 2. The numbers for EN and KRR refer to an average of 20/5 (EN/KRR) times random shuffling with training/test proportion of 90%/10%. The accuracy achieved on PC9 by all models is lower compared to the one achieved on QM9 data. This result could derive from higher chemical homogeneity of QM9 and a more curated workflow of quantum computational calculations (see "Chemical differences between QM9 and PC9" section). For KRR, the performances are quite disappointing even for the Molecular Orbitals energies. In Fig. 2 the learning curves for total SCF energies E of SchNet and KRR models with Coulomb matrices are reported for both datasets. When the training set encompasses more than 10000 compounds the NN models become competitive. The performance of SchNet on a new dataset is promising. The PC9 dataset appears to be a bit more challenging than QM9.

### Generalization ability of SchNet neural networks models

Since the QM9 and PC9 are quite comparable in size, we were able to test the generalizability of the SchNet models.

#### Predicting PC9 with QM9 models

**Fig. 3:**
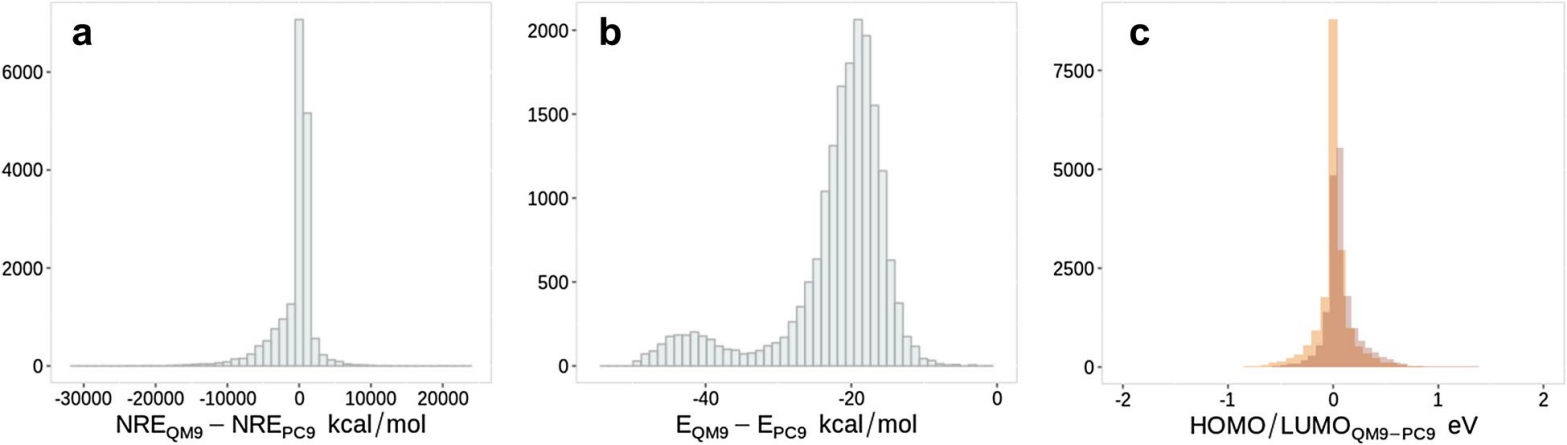
Histogram of distribution of the difference in **a** total energies E, **b** in Nuclear Repulsion Energies (NRE), **c** in HOMO (darker) and LUMO energies for 18,357 molecules present in both QM9 and PC9

**Fig. 4:**
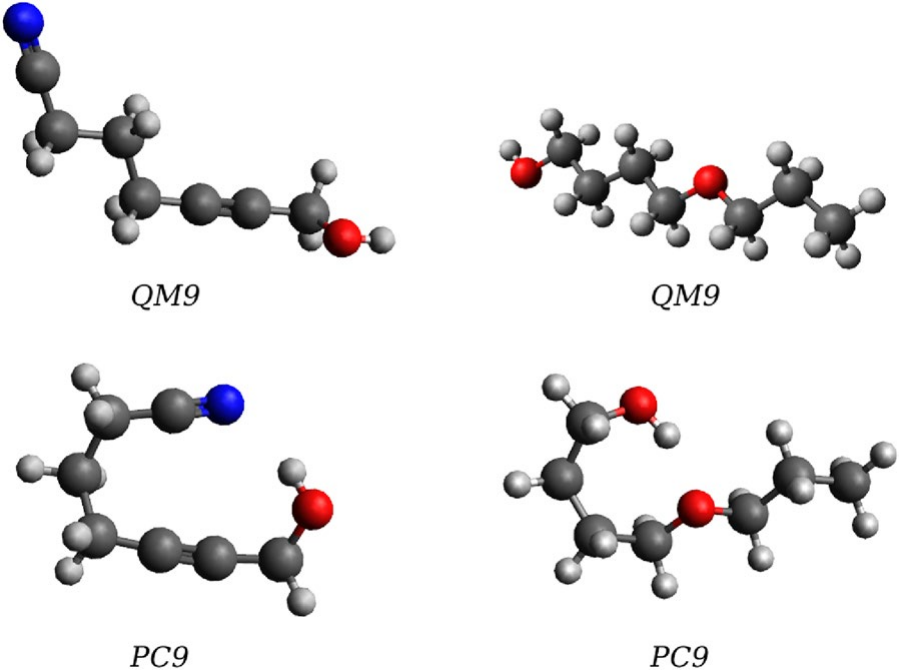
Examples of structures with large difference in Nuclear Repulsion Energies (NRE) calculated for QM9 and PubChemQC geometry

The SchNet model trained on QM9 data has been used to predict two subsets of PC9. The first one (A) is a subset which is common for both QM9 and PC9 (see "Reproducibility of QM9") and the second subset (B) consists of compounds that did not appear in QM9. However, the molecules in subset A are not identical. The differences in energies and in nuclear repulsion energy (NRE) between the corresponding compounds from QM9 and from PC9 are shown in Fig. 3a, b. Some compounds present a huge NRE difference. That is due to InChI comparison that could match different tautomeric or conformational forms. Figure 4 demonstrates the examples with $\Delta$NRE >10,000 kcal/mol). Due to the slightly different level of theory, total energies of QM9 are also consistently lower compared to energies of PC9 (Fig. 3b), with a maximum at − 20 kcal/mol. But, a linear relationship could compensate the divergence caused by different basis sets (B3LYP/6-31G(2df,p) *vs.* B3LYP/6-31G(d)). Once the energetic values of PC9 have been predicted by the model, we used an a posteriori linear correction to compare them with the original DFT calculated energies. The best fit has been obtained with the Huber regression linear models [50]. The fitted values were then compared with the original values. It is worth noting here that this regression is essentially useful for total molecular energies since the differences in HOMO and LUMO energies between QM9 and PC9 are centered at 0 eV (see Fig. 3c).

**Fig. 5:**
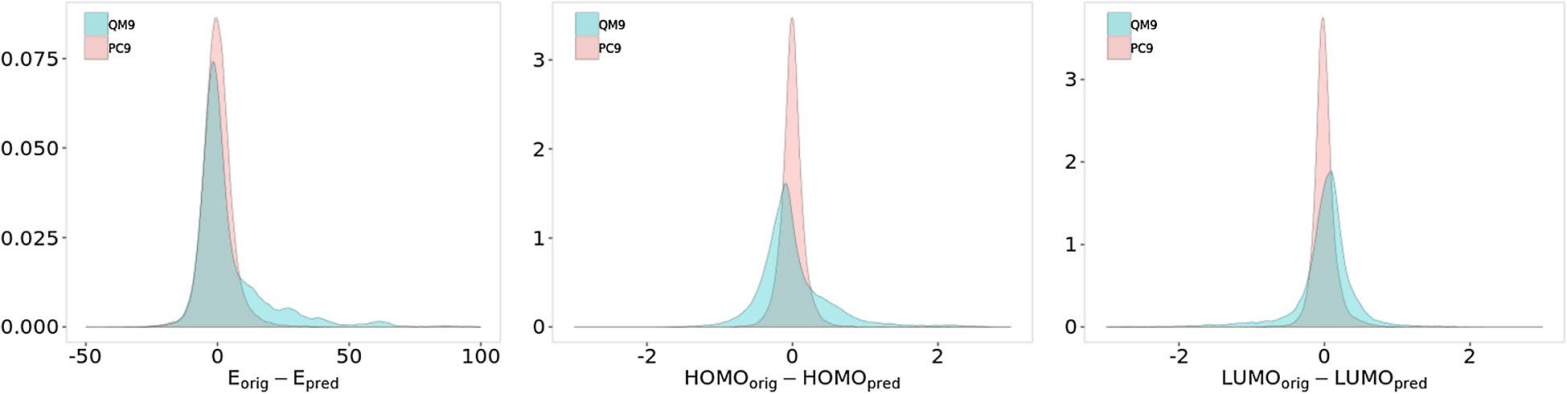
Generalization ability results: error density plots for the prediction of total energies E (kcal/mol), HOMO and LUMO energies (eV) of set B. The blue (pink) curve represents predictions by a model trained on QM9 (PC9) on unknown molecules exclusive to PC9 (QM9)

*Performance on E*, $\epsilon _{(HOMO)}$ *and* $\epsilon _{(LUMO)}$. The results for the predictions of the three properties on subsets A and B after linear correction are reported in Table 2. For E, a MAE of 3.0 kcal/mol is found on the subset A. It is more than the 1.0 kcal/mol found on QM9 when trained on QM9. But, an increase in MAE was expected since the QM9-PC9 energy differences has been found to be quite spread (see Fig. 3b). The predictions are above the chemical accuracy but still much better than a KRR prediction on PC9, trained on PC9. However, the prediction of E on subset B gives a MAE of 8.9 kcal/mol. Figure 5 shows the density plot for subset B predictions. For clarity reasons, we have cut the x-axis range of the graph from −50 to +100 kcal/mol. Some 231 extreme and spread outliers were omitted by this way (225 of QM9 model and 6 of PC9 model). The blue color represents the results obtained with the model trained on QM9 and applied on subset B of PC9. The plot for total energies has a clear bias toward overestimated energy of certain part of data. An analysis of the outliers follows in the next paragraph. Concerning the results for HOMO and LUMO energies predictions, the MAE for subset A are almost the same as observed on the training set (see Table 2). The density plots for subset B correspond to spread and asymmetrical curves (Fig. 5, blue), associated with MAE of 0.33 and 0.27 eV.

*Outliers analysis.* To propose a rationale for the observed degraded performance, the species with an absolute prediction error over 30 kcal/mol for E and 1.5 eV for HOMO and LUMO energies were extracted and analyzed. Out of 80,877 compounds of subset B, 5,305 (6.5%) present MAE over 30 kcal/mol on E and 1527 (1.88%) present MAE over 1.5 eV on HOMO energy. They can be classified mainly into two main classes: (1) outliers with multiplicity different than one and (2) outliers with specific functional groups

**Fig. 6:**
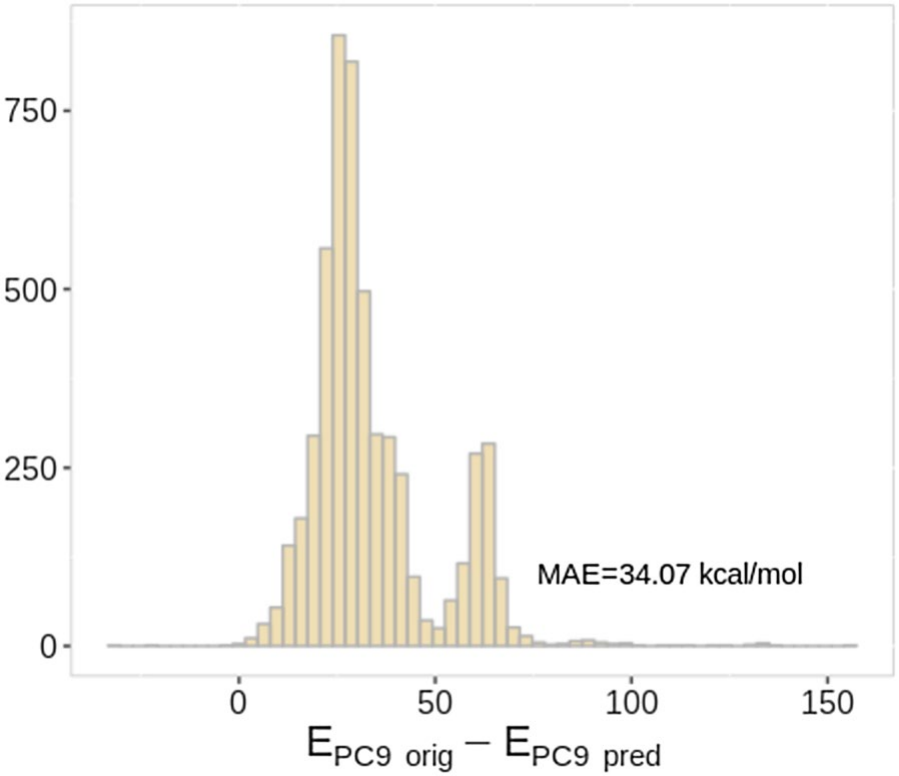
Original vs predicted by QM9 SchNet model total molecular energies (E, kcal/mol) of PC9 molecules with multiplicity equal to two or three

As it was mentioned earlier (see "PubChemQC subset: PC9"), PC9 contains compounds with multiplicities ranging from one to three whereas QM9 data are strictly closed shelled systems. Radicals and triplets possess indeed electronic structures that are usually more reactive. Out of 5,325 PC9 compounds with a multiplicity not equal to one, 2,476 were outliers. Among them, there were 883 molecules bearing multiplicity of 3, which is 100% of all the triplet compounds. 35.5% of all monoradicals are outliers in PC9. The histogram of the original and predicted total energies for the 5,325 molecules with non single multiplicity is shown in Fig. 6. It can be observed that the neural network always overestimates the stability of such compounds, since not provided the information about the multiplicity. For the HOMO energies, out of 1527 outliers, 1200 were attributed to molecules with the multiplicity> 1.

The next class of structures with large prediction errors includes molecules with specific functional groups. Even with the same atom list (H, C, N, O and F), the chemical diversity of QM9 and PC9 are different (see Table 3 and "Chemical differences between QM9 and PC9" section). Three chemical functions, determined by Checkmol software, have been constantly spotted among outliers [51]. These were diaryl ethers (0.01% of PC9 and absent in QM9), peroxydes (0.17% of PC9 and absent in QM9) and dyarilamines (very few cases in PC9 and absent in QM9). Out of 5,305 outliers, 1,320 belong to one of these three classes. Finally, it could be noted that 568 structures of the outliers present rare functional groups that were not detected by Checkmol (such as peroxyacetylnitrates, diflouroamines, perfluoroallenes, nitramines, tetraenes...).

**Table 3:** Selection of functional groups detected by Checkmol with their corresponding number of molecules in PC9 and QM9 datasets and MAE in generalization conditions

Selection	Functional group classes	PC9 data		QM9 data	
		Occurrences	QM9 model	Occurrences	PC9 model
CF and NN bonds	Azide	358	11.5	0	–
	Azo compound	272	5.3	10	4.1
	Acyl fluoride	105	6.4	0	–
	Aryl fluoride	936	9.4	1562	4.1
	Alkyl fluoride	4576	6.8	52	4.3
Abundant in QM9	Carbonitrile	4624	5.4	*10315*	4.7
	Secondary alcohol	6282	6.2	*10668*	4.3
	Trialkylamine	3301	6.4	*10687*	4.2
	Alkyne	3906	6.1	*10873*	4.5
	Tertiary amine	3388	6.4	*11057*	4.2
	Aromatic compound	12728	8.2	*15863*	4.3
	Dialkyl ether	9275	6.2	*24012*	4.3
	Heterocyclic compound	42665	7.0	*61904*	4.3
QM9 model focus	Hydroperoxide	717	*45.4*	0	–
	Diaryl ether	22	*39.1*	0	–
	Peroxide	430	*32.7*	0	–
	Diarylamine	11	*32.6*	0	–
	Carbamic acid halide	16	*24.4*	0	–
	Nitrite	1	*22.7*	0	–
	Hydroxamic acid	46	*19.4*	0	–
	Nitroso compound	48	*17.0*	0	–
	Hydroxylamine	805	*16.2*	13	3.4
	Hemiacetal	819	*2.8*	0	–
PC9 model focus	Enol ether	1977	4.4	2	*16.0*
	Amide acetal	59	14.2	28	*7.1*
	Carboxylci acid	4213	6.6	106	*6.2*
	Hemiaminal	3034	6.5	207	*6.0*
	Acyl cyanide	67	4.0	281	*6.0*

The selection italic CF and NN interactions, the most prominent groups in QM9, the largest MAE of the model trained on QM9 and the largest MAE of the model trained on PC9

#### Predicting QM9 with PC9 model

In a reciprocal approach, the SchNet NN trained on PC9 data has been used for the prediction of QM9. Two subsets of QM9 have been prepared, subset A with the molecules common for both databases (18,357) and subset B (110,286) with the molecules exclusive to QM9. Subset A is therefore the same for QM9 and PC9, but their modeled values and their geometries are different with regard to the basis set. To compensate the basis set effect and to fit the predicted *vs* original values of QM9, the best correction was also found with an *a posteriori* Huber linear regression models [52].

Table 2 shows the results of the prediction for the two subsets. For subset A, the results obtained by PC9 model are similar to the subset A results with the QM9 model. With 3.0 compared to 3.4 kcal/mol for E and 0.07/0.06 compared to 0.05/0.05 for HOMO and LUMO energies, the two models can therefore transfer their training on familiar data. But the most interesting result is the ability of PC9 model to predict unfamiliar data with a minimal loss of precision (4.2 compared to 3.4 kcal/mol for E in contrast to QM9 model with 8.9 and 3.0 kcal/mol). The accuracy for the HOMO and LUMO energies is almost at the 0.1 eV goal. Figure 5 demonstrates the generalization ability of the model trained on PC9 over the subset B of QM9 (in pink) compared with the performance of QM9 model on subset B of PC9 (in blue). Indeed, error density plots of PC9 model are much narrower than their QM9 counterparts, indicating that there is not much of QM9 that is unfamiliar to PC9. This fact could speak for better generalization ability of PC9 models due to higher chemical diversity or even fewer purity or organization of data, which to a certain limit could make the model more stable toward noise and over-fitting.

### Chemical differences between QM9 and PC9

#### Bond distances analysis

**Fig. 7:**
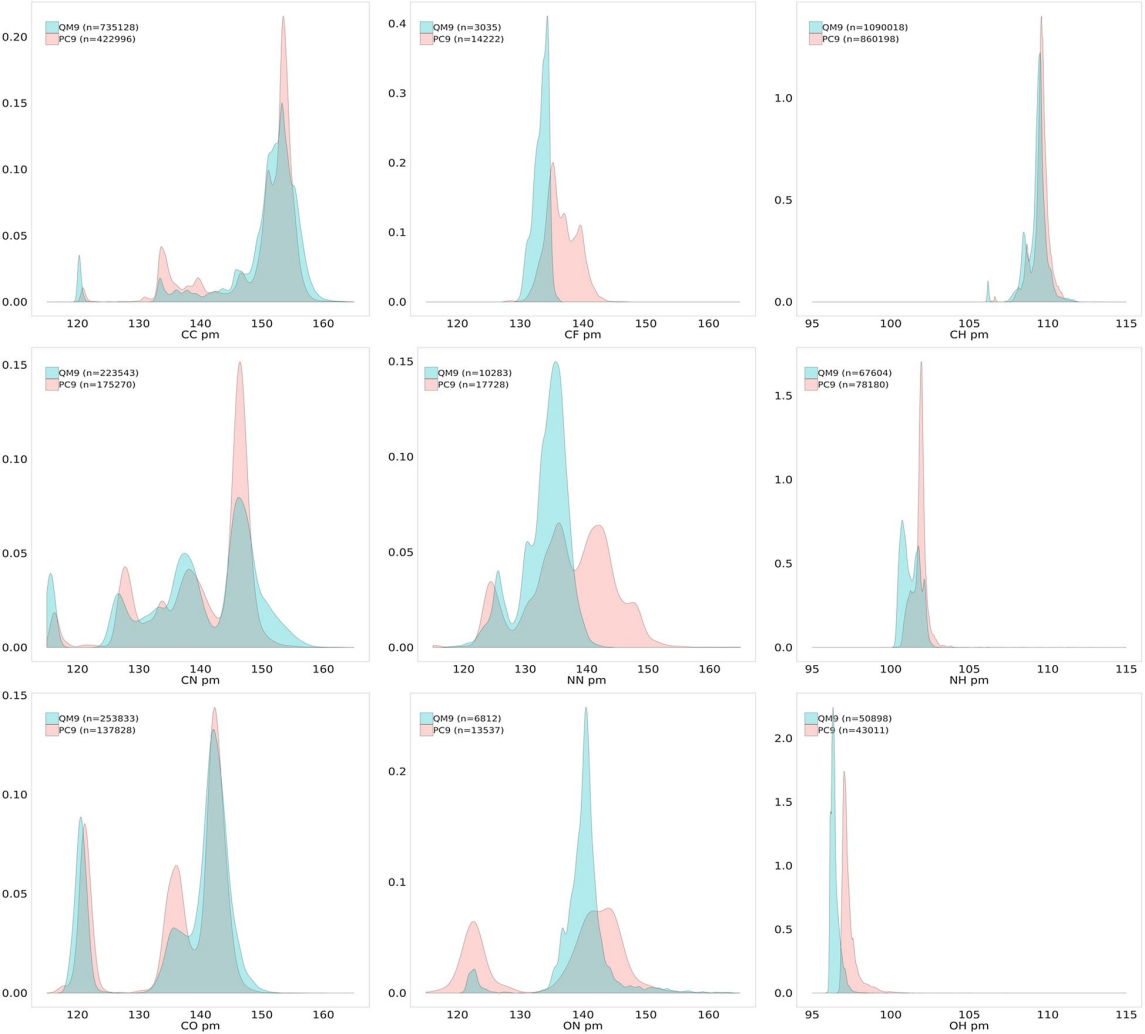
Density distribution of pairwise bond lengths in QM9 and PC9

A way of looking at chemical diversity could be a pairwise analysis of bond length distributions over the two datasets. It has been done for the following pairs: C–C, C–H, C–N, C–O, C–F, N–N, N–H, O–N and O–H. Density distributions of those pairs is represented in Fig. 7. There are clearly more alkynes (CC distance around 120 pm) in QM9. The distribution of C–H bonds is identical for both datasets and the slight shift can be assigned to the difference in the basis sets. An interesting observation could be found for the C–F and N–N bond distributions. In both cases the density of QM9 is located to the left. The fact that PC9 shows a much broader range of interactions designates that PC9 encompasses a more diverse chemistry.

#### Functional groups analysis

Therefore, an analysis of chemical composition and chemical diversity of QM9 and PC9 has been carried out. The functional group analysis has been achieved with the Checkmol software, capable of recognising 200 different functional groups [51]. The difference in the number of identified functional groups between QM9 and PC9 is quite substantial with 97 groups spotted at least once in PC9 for 71 groups in QM9. Furthermore, the average number of functional groups per molecule is also different with 2.0 chemical group per molecule in PC9 compared to 3.1 in QM9. QM9 data therefore presents less diversity but also a higher concentration of chemical functions in only 9 “heavy” atoms at most. The functional groups were explicitly added during the molecular generation in QM9 to an extend that exceeds a real-life dataset.

**Fig. 8:**
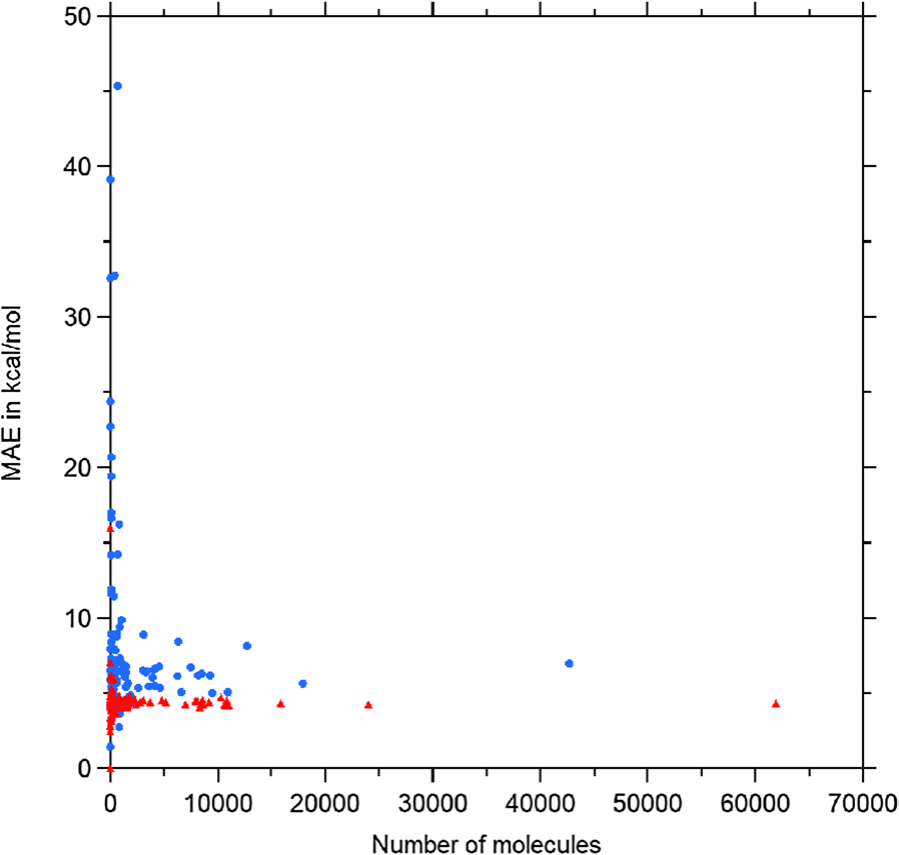
Generalization ability results: Mean absolute error for each functional groups subsets vs the number of molecules with the functional group on the test set. The blue dots (red triangles) represent predictions by a model trained on QM9 (PC9) on the other dataset PC9 (QM9)

A list of the number of molecules in QM9 and PC9 with specific groups is given in Table 3. The complete table is available as an additional file. Different kinds of amines, alcohols, heterocycles, ether and aromatic compounds form the largest classes for both datasets. However, the table emphasizes MAE per functional group. 12 of the 16 functional groups with MAE over 10 kcal/mol on PC9 data for the model trained on QM9 are totally absent from QM9. It is the case of hydroperoxide, diarylether, peroxide, diaryl amine, nitrite, nitroso, cyanate... Keep in mind that small subsets predictions (molecules with specific uncommon functional groups) will be also more affected by outliers. Nevertheless, the Table 3 and the Fig. 8 show that many unseen functional groups can also be correctly predicted by QM9 model like the hemiacetal. There is clearly some knowledge transfer. The model trained on PC9 shows a much homogeneous description of all functional groups. Apart from the 2 molecules with an enol ether function in QM9 (MAE > 10 kcal/mol), the biggest error is 7.1 kcal/mol for the amide acetal.

#### Scaffold analysis

**Fig. 9:**
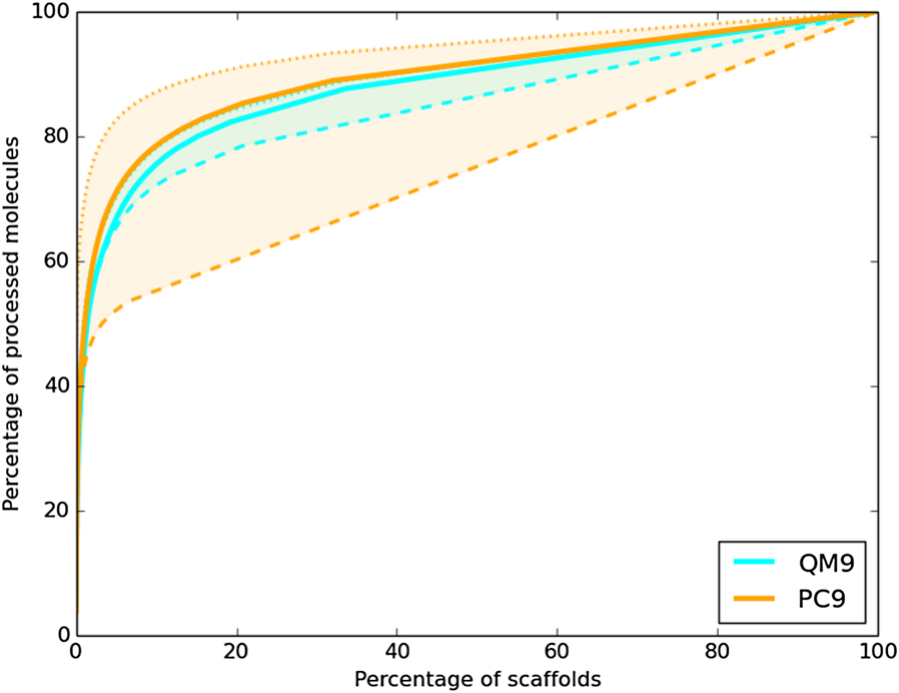
Cumulative scaffold frequency plots of Level 1 scaffolds of the QM9 and PC9 datasets

Chemical diversity has also been studied by the mean of scaffolds analysis [53–59]. We have used Scaffold network generator to generate the first layer of scaffolds for both datasets [56]. Those scaffolds correspond to (poly)cyclic core. However, there are two issues for such analysis on QM9 and PC9. Firstly, QM9 and PC9 are limited up to nine “heavy” atoms. Many graph frameworks are missing compared to the ones found in organic chemistry by Lipkus et al. or in the PubChem analysis by Velkoborsky et al. Secondly, the ratio of acyclic compounds is radically different in the two datasets. In QM9, 10.5% of the molecules are acyclic and 24844 different scaffolds are found on the rest . For PC9, 40% of the molecules are acyclic and 11883 different scaffolds are found on the rest. QM9 does present much more scaffolds than PC9 with more complex polycyclic architectures. But, as it has been observed with the bond length analysis, QM9 lacks chemical diversity in the acyclic part. To resume the scaffold analysis, their cumulative frequency plots are represented in Fig. 9. The solid lines correspond to the compounds with a cyclic core. Both curves are straight lines for the last two thirds of the scaffolds. That means that both datasets are mainly composed of singletons (unique graph frameworks). In fact, for both datasets the most abundant core scaffold is the three member ring C1CC1 (4.8% in QM9 and 3.7% in PC9). In Fig. 9, we have also tried to put in perspective the missing acyclic chemical diversity. The dashed and dotted lines represent the two limit cases where all the acyclic compounds are considered in the same scaffold (upper limit) and where all the acyclic compounds are considered in unique scaffolds (lower limit). It appears that a scaffold analysis seems to be quite unreliable with a high ratio of acyclic molecules as it is the case for PC9.

#### Self organizing maps analysis

**Fig. 10:**
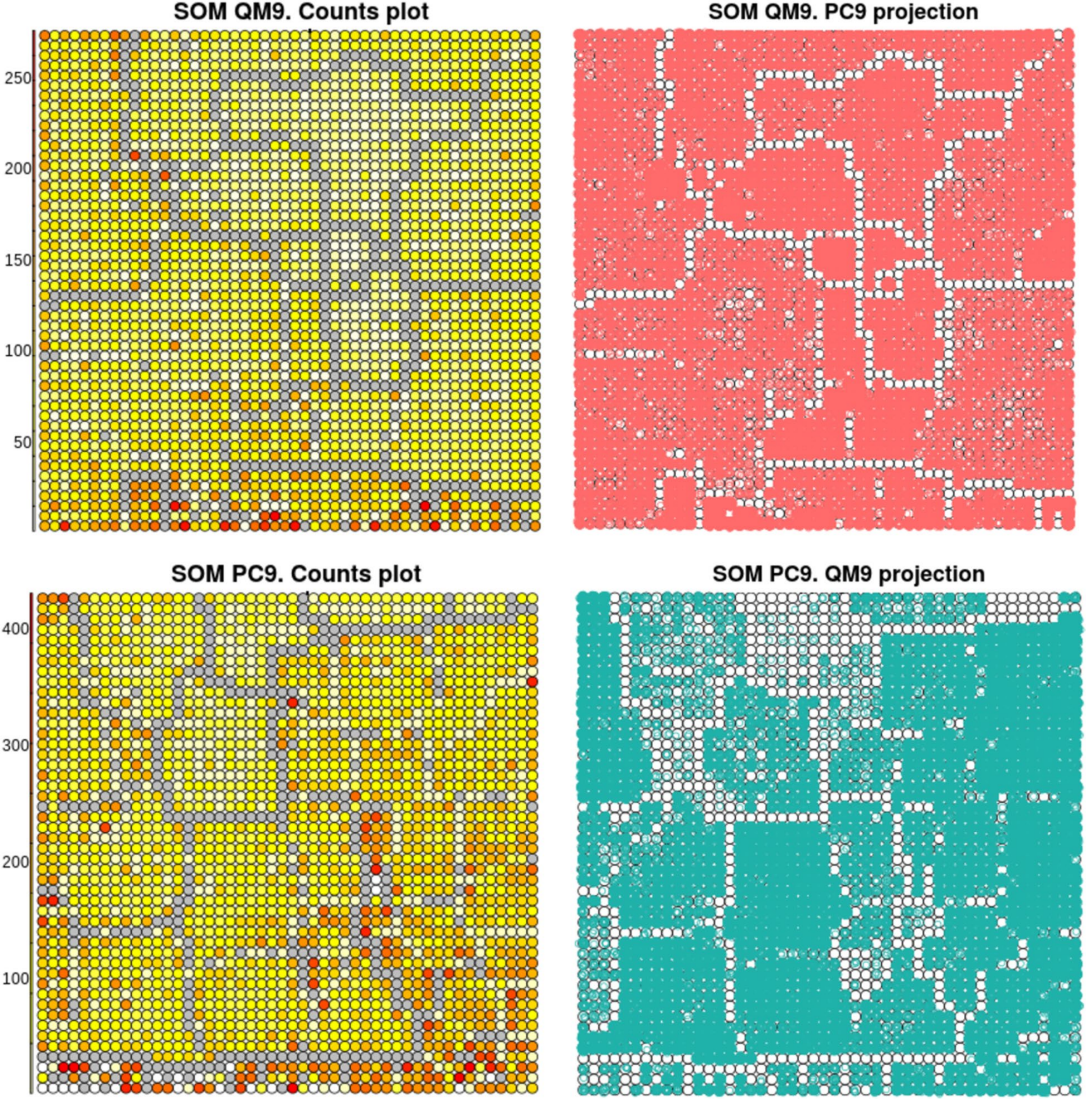
Self Organizing Maps based on the Coulomb matrices of the QM9 and PC9 datasets (grey = empty dots, red = most crowded dots) and the corresponding projections of the other dataset

We have used Self Organizing Maps (SOM) to visually represent a chemical space divergence between QM9 and PC9. R ’kohonen’ package has been used to produce a 2D discrete map with the Coulomb matrices as input [60]. Grids were set to 50x50 for QM9 and 48x48 for PC9. Default parameters have been used. In Fig. 10, the left maps represent the obtained organization with red dots corresponding to higher data concentration and grey points indicating empty regions. Taking into account the scale of nodes occurrence, one can notice that PC9 SOM has more overpopulated regions compared to QM9 SOM. Data distribution of the latter is more uniform, which speaks for higher consistency in chemical classes ratio in QM9. The right maps of Fig. 10 represent the projection of another data. In the upper right, the projected PC9 data is evenly spread onto the QM9 map, whereas in the lower right, the QM9 data leaves many nodes of PC9 map empty. By this way we can conclude that QM9 is lacking some chemistry included in PC9. However, that does not prove that PC9 possesses the whole chemical diversity associated with H, C, N, O and F.

Furthermore, we propose here a formula to quantify the *affinity* of a projected dataset B onto a SOM built on the other dataset A. It accounts for two terms. The first term captures the similarity of molecular density distribution over the SOM, expressed as $e^{-\left| p_{A,i} - p_{B,i} \right| }$, where $p_{A,i}$ (resp. $p_{B,i}$) is the proportion of molecules in node *i* for the set A (resp. B). The second term captures the degree of similarity in distances between the two sets in a node. This term as well reflects a chemical resemblance of molecules in set A and B since molecules of same chemical class/cluster would have analogous distances. This last term is expressed as $e^{-(\left| \overline{d_{A,i}} - \overline{d_{B,i}} \right| /(dmax_{A,i}-dmin_{A,i}))}$, where $\overline{d_{A,i}}$ (resp. $\overline{d_{B,i}}$) is the average distance of molecules in a node *i* for the set A (resp. B) and $dmax_{A,i}$ (resp. $dmin_{A,i}$) is a maximal (resp. minimal) distance of molecules in the node *i* for the set A. (See Additional files: 1, 2 and 3 for more details).

$$\begin{aligned} S_a= \sum _{i=1}^{Nnodes}p_{B,i} \times e^{- \left( \left| p_{A,i} - p_{B,i} \right| + \frac{\left| \overline{d_{A,i}} - \overline{d_{B,i}}\right| }{dmax_{A,i}-dmin_{A,i}}\right) } \end{aligned}$$

This score ranges from 0 to 1, where 0 means that the affinity of the projected data B to the SOM built on data A is very low. According to the calculated affinity scores reported in the Table 4, the density term is very similar for both projections meaning that both SOMs are proportionally distributed. However, the distance term of QM9 SOM is lower compared to PC9 SOM. Fewer diversity of functional groups of QM9 leads to a less universal SOM, upon which the PC9 molecules of uncommon classes would be projected mixed with the known classes. That will lead to lower chemical purity per a node and decrease the distance term.

**Table 4:** Affinity score of PC9 and QM9 data projected onto the other dataset SOM

	$S_a$	Density	Distance
PC9 data on QM9 SOM	0.8456	0.9973	0.8480
QM9 data on PC9 SOM	0.9370	0.9989	0.9381

The affinity score, the density term and the distance term are defined in "Self organizing maps analysis" section

## Conclusion

Machine Learning models are able to predict molecular properties such as total molecular energies and frontier molecular orbitals energies in a reproducible way and within chemical accuracy. But, those ML models are sensitive to setup of hyperparameters. We have managed to tune those correctly in order to reproduce the literature results for EN, KRR (with Coulomb matrices) and SchNet neural networks, albeit with the help from the authors of the models. Thus, reproduction of such results is time consuming.

We have shown here that an ML model able to be generalized with enough accuracy on unknown molecules requires to be trained on a wide chemical diversity. The golden standard dataset QM9 is limited to H, C, N, O and F but still lacks chemical functional groups associated with this list of atoms. A new dataset, PC9, is presented here. It has been extracted from the PubChemQC data, and mimic the QM9 limitations (same atom types and size limit). Comparable in size, we have found that only 18% of PC9 is common with QM9. More importantly, we managed to study the generalization ability of the built SchNet models for each dataset by means of prediction of molecules from the other dataset, despite slightly different levels of quantum theory. We found that models trained on PC9 demonstrate better generalization ability than the models trained on QM9. It is related both to the presence of radicals, triplets and more functional groups.

This work highlights the crucial problem of chemical diversity in standard datasets. Checking every functional groups in every molecules allowed for a list of under represented functions in QM9. We found that such under representation in the training data is indeed a major cause of outliers in predictions. Going through all bonding distances pairs in the datasets confirmed a tangible chemical divergence between QM9 and PC9, especially for C–F, N–N and N–O. A scaffold analysis showed a larger collections of graph frameworks in QM9 and high ratio of acyclic compounds in PC9. Finally, a visual chemical space analysis was preformed with Self Organising Map, revealing the presence of zones in PC9 chemical space not occupied by QM9 data, contrary to the absence of those in case of the projection of QM9 onto PC9 map. To quantify the affinity of a projected data for a SOM, we have proposed a novel affinity index. Its value for PC9 SOM is higher than for QM9 SOM and driven mainly by the difference in distances between projected and initial data. SOM is one of the visualization tool but a real quantification method of the chemical diversity is still an open problem and a key point for an automatic generation of a dataset that maximize chemical diversity.

## Supplementary information

**Additional file 1.** AML building example. Source code for the ML results.

**Additional file 2.** Functional groups analysis. Complete table of all encountered functional groups in PC9 and QM9.

**Additional file 3.** Affinity index. Details on the SOM affinity analysis.

## Data Availability

The PC9 dataset has been shaped to mimic the QM9 dataset and has been deposited in the public repository figshare with DOI: 10.6084/m9.figshare.9033977

## References

[1] Smith JS, Nebgen BT, Zubatyuk R, Lubbers N, Devereux C, Barros K, Tretiak S, Isayev O, Roitberg AE (2019). Approaching coupled cluster accuracy with a general-purpose neural network potential through transfer learning. Nat Commun.

[2] Unke OT, Meuwly M (2019). PhysNet: a neural network for predicting energies, forces, dipole moments, and partial charges. J Chem Theory Comput.

[3] Wilkins DM, Grisafi A, Yang Y, Lao KU, DiStasio RA, Ceriotti M (2019). Accurate molecular polarizabilities with coupled cluster theory and machine learning. Proc Natl Acad Sci.

[4] Iype E, Urolagin S (2019). Machine learning model for non-equilibrium structures and energies of simple molecules. J Chem Phys.

[5] Duan C, Janet JP, Liu F, Nandy A, Kulik HJ (2019). Learning from failure: predicting electronic structure calculation outcomes with machine learning models. J Chem Theory Comput.

[6] Grisafi A, Fabrizio A, Meyer B, Wilkins DM, Corminboeuf C, Ceriotti M (2019). Transferable machine-learning model of the electron density. ACS Cent Sci.

[7] Okamoto Y (2019). Data sampling scheme for reproducing energies along reaction coordinates in high-dimensional neural network potentials. J Chem Phys.

[8] Chandrasekaran A, Kamal D, Batra R, Kim C, Chen L, Ramprasad R (2019). Solving the electronic structure problem with machine learning. NPJ Comput Mater.

[9] Amabilino S, Bratholm LA, Bennie SJ, Vaucher AC, Reiher M, Glowacki DR (2019). Training neural nets to learn reactive potential energy surfaces using interactive quantum chemistry in virtual reality. J Phys Chem A.

[10] Cheng L, Welborn M, Christensen AS, Miller TF (2019). A universal density matrix functional from molecular orbital-based machine learning: transferability across organic molecules. J Chem Phys.

[11] Ghosh K, Stuke A, Todorović M, Jørgensen PB, Schmidt MN, Vehtari A, Rinke P (2019). Deep learning spectroscopy: neural networks for molecular excitation spectra. Adv Sci.

[12] Rupp M, Tkatchenko A, Müller K-R, von Lilienfeld OA (2012). Fast and accurate modeling of molecular atomization energies with machine learning. Phys Rev Lett.

[13] Hansen K, Montavon G, Biegler F, Fazli S, Rupp M, Scheffler M, von Lilienfeld OA, Tkatchenko A, Müller K-R (2013). Assessment and validation of machine learning methods for predicting molecular atomization energies. J Chem Theory Comput.

[14] Hansen K, Biegler F, Ramakrishnan R, Pronobis W, von Lilienfeld OA, Müller K-R, Tkatchenko A (2015). Machine learning predictions of molecular properties: accurate many-body potentials and nonlocality in chemical space. J Phys Chem Lett.

[15] Ramakrishnan R, von Lilienfeld OA (2015) Many molecular properties from one Kernel in chemical space. arXiv:1502.04563 [physics.chem-ph], 140022 10.2533/chimia.2015.182 26672132

[16] Huang B, von Lilienfeld OA (2016). Communication: understanding molecular representations in machine learning: the role of uniqueness and target similarity. J Chem Phys.

[17] Faber FA, Hutchison L, Huang B, Gilmer J, Schoenholz SS, Dahl GE, Vinyals O, Kearnes S, Riley PF, von Lilienfeld OA (2017). Prediction errors of molecular machine learning models lower than hybrid DFT error. J Chem Theory Comput.

[18] Collins CR, Gordon GJ, von Lilienfeld OA, Yaron DJ (2018). Constant size descriptors for accurate machine learning models of molecular properties. J Chem Phys.

[19] Bartók AP, De S, Poelking C, Bernstein N, Kermode JR, Csányi G, Ceriotti M (2017). Machine learning unifies the modeling of materials and molecules. Sci Adv.

[20] Pereira F, Xiao K, Latino DARS, Wu C, Zhang Q, Aires-de-Sousa J (2017). Machine learning methods to predict density functional theory B3lyp energies of HOMO and LUMO orbitals. J Chem Inform Model.

[21] Montavon G, Rupp M, Gobre V, Vazquez-Mayagoitia A, Hansen K, Tkatchenko A, Müller K-R, Anatole von Lilienfeld O (2013). Machine learning of molecular electronic properties in chemical compound space. New J Phys.

[22] Smith JS, Isayev O, Roitberg AE (2017). ANI-1: an extensible neural network potential with DFT accuracy at force field computational cost. Chem Sci.

[23] Gilmer J, Schoenholz SS, Riley PF, Vinyals O, Dahl GE (2017) Neural message passing for quantum chemistry. arXiv:1704.01212 [cs]

[24] Schütt KT, Sauceda HE, Kindermans P-J, Tkatchenko A, Müller K-R (2018). SchNet—a deep learning architecture for molecules and materials. J Chem Phys.

[25] Hy TS, Trivedi S, Pan H, Anderson BM, Kondor R (2018). Predicting molecular properties with covariant compositional networks. J Chem Phys.

[26] Hou F, Wu Z, Hu Z, Xiao Z, Wang L, Zhang X, Li G (2018). Comparison study on the prediction of multiple molecular properties by various neural networks. J Phys Chem A.

[27] Lubbers N, Smith JS, Barros K (2018). Hierarchical modeling of molecular energies using a deep neural network. J Chem Phys.

[28] Unke OT, Meuwly M (2018). A reactive, scalable, and transferable model for molecular energies from a neural network approach based on local information. J Chem Phys.

[29] Bartók AP, Kondor R, Csányi G (2013). On representing chemical environments. Phys Rev B.

[30] Willatt MJ, Musil F, Ceriotti M (2018). Feature optimization for atomistic machine learning yields a data-driven construction of the periodic table of the elements. Phys Chem Chem Phys.

[31] Faber FA, Christensen AS, Huang B, von Lilienfeld OA (2018). Alchemical and structural distribution based representation for universal quantum machine learning. J Chem Phys.

[32] Ramakrishnan R, Dral PO, Rupp M, von Lilienfeld OA (2014). Quantum chemistry structures and properties of 134 kilo molecules. Sci Data.

[33] Ruddigkeit L, van Deursen R, Blum LC, Reymond J-L (2012). Enumeration of 166 billion organic small molecules in the chemical universe database GDB-17. J Chem Inform Model.

[34] Schütt KT, Arbabzadah F, Chmiela S, Müller KR, Tkatchenko A (2017). Quantum-chemical insights from deep tensor neural networks. Nat Commun.

[35] Schütt KT, Kindermans P-J, Sauceda HE, Chmiela S, Tkatchenko A, Müller K-R (2017) SchNet: A continuous-filter convolutional neural network for modeling quantum interactions. arXiv:1706.08566 [physics, stat]

[36] Wang Y, Xiao J, Suzek TO, Zhang J, Wang J, Bryant SH (2009). PubChem: a public information system for analyzing bioactivities of small molecules. Nucleic Acids Res.

[37] Kim S, Chen J, Cheng T, Gindulyte A, He J, He S, Li Q, Shoemaker BA, Thiessen PA, Yu B, Zaslavsky L, Zhang J, Bolton EE (2019). PubChem 2019 update: improved access to chemical data. Nucleic Acids Res.

[38] Nakata M, Shimazaki T (2017). PubChemQC project: a large-scale first-principles electronic structure database for data-driven chemistry. J Chem Inform Model.

[39] Heller SR, McNaught A, Pletnev I, Stein S, Tchekhovskoi D (2015). InChI, the IUPAC international chemical identifier. J Cheminform.

[40] O’Boyle NM, Banck M, James CA, Morley C, Vandermeersch T, Hutchison GR (2011). Open babel: an open chemical toolbox. J Cheminform.

[41] Pedregosa F, Varoquaux G, Gramfort A, Michel V, Thirion B, Grisel O, Blondel M, Prettenhofer P, Weiss R, Dubourg V, Vanderplas J, Passos A, Cournapeau D (2005). Scikit-learn: machine learning in Python. J Mach Learn Res.

[42] Zou H, Hastie T (2005). Regularization and variable selection via the elastic net. J R Stat Soc.

[43] Li F, Yang Y, Xing EP (2005) From lasso regression to feature vector machine. In: Proceedings of the 18th international conference on neural information processing systems. NIPS’05, pp. 779–786. MIT Press, Cambridge, MA. http://dl.acm.org/citation.cfm?id=2976248.2976346

[44] Hoerl AE, Kannard RW, Baldwin KF (1975). Ridge regression: some simulations. Commun Stat.

[45] Haykin SS (2009). Neural networks and learning machines.

[46] Mallat S (2016). Understanding deep convolutional networks. Philos Trans R Soc A.

[47] Glorot X, Bengio Y (2010) Understanding the difficulty of training deep feedforward neural networks. In: Teh YW, Titterington M (eds) Proceedings of the thirteenth international conference on artificial intelligence and statistics. Proceedings of machine learning research, vol 9, pp 249–256. PMLR, Chia Laguna Resort, Sardinia. http://proceedings.mlr.press/v9/glorot10a.html

[48] Schmidhuber J (2015). Deep learning in neural networks: an overview. Neural Netw.

[49] Schütt KT, Kessel P, Gastegger M, Nicoli KA, Tkatchenko A, Müller K-R (2019). SchNetPack: a deep learning toolbox for atomistic systems. J Chem Theory Comput.

[50] Huber regression parameters of QM9 model on PC9. For E and subset A: Orig = 0.9999315933 * fit -0.0000040081, For E and subset B: Orig = 0.9999188417 * fit -0.0000040914

[51] Haider N (2010). Functionality Pattern Matching as an Efficient Complementary Structure/Reaction Search Tool: an Open-Source Approach. Molecules.

[52] Huber regression parameters of PC9 model on QM9. For E and subset A: Orig = 23.0616524941 * fit -0.1115732596, For E and subset B: Orig = 23.0616907882 * fit -0.1735192942

[53] Lipkus AH, Yuan Q, Lucas KA, Funk SA, Bartelt WF, Schenck RJ, Trippe AJ (2008). Structural diversity of organic chemistry. A scaffold analysis of the CAS registry. J Org Chem.

[54] Wetzel S, Klein K, Renner S, Rauh D, Oprea TI, Mutzel P, Waldmann H (2009). Interactive exploration of chemical space with Scaffold Hunter. Nat Chem Biol.

[55] Hu Y, Stumpfe D, Bajorath J (2011). Lessons learned from molecular scaffold analysis. J Chem Inform Model.

[56] Matlock MK, Zaretzki JM, Swamidass SJ (2013). Scaffold network generator: a tool for mining molecular structures. Bioinformatics.

[57] González-Medina M, Prieto-Martínez FD, Owen JR, Medina-Franco JL (2016). Consensus diversity plots: a global diversity analysis of chemical libraries. J Cheminform.

[58] Velkoborsky J, Hoksza D (2016). Scaffold analysis of PubChem database as background for hierarchical scaffold-based visualization. J Cheminform.

[59] Shang J, Sun H, Liu H, Chen F, Tian S, Pan P, Li D, Kong D, Hou T (2017). Comparative analyses of structural features and scaffold diversity for purchasable compound libraries. J Cheminform.

[60] Wehrens R, Kruisselbrink J (2018). Flexible self-organizing maps in kohonen 3.0. J Stat Softw.

