# Supplementary material for: Dataset’s chemical diversity limits the generalizability of machine learning predictions
Source: J Cheminform. 2019 Nov 12;11:69. doi: 10.1186/s13321-019-0391-2 (PMC6852905; doi:10.1186/s13321-019-0391-2)
Supplement: Supplementary file 3 — Additional file 3. Affinity index. Details on the SOM affinity analysis. [file 13321_2019_391_MOESM3_ESM.pdf]

Glavatskikh Marta
30.08.2019

SOM projection affinity index

Goal: to provide a quantitative measure of the projection quality of data B onto the SOM built on data A.

Issue: the reported measurements concerning 2D data distribution are revolving around clustering problem, i.e how to optimally measure the degree of segregation of several clusters on the map. Those formulas do not fit our task since the characteristic we would like to estimate is basically the *compatibility* of the projected data to the map built on another data.

Proposition: The available information associated with a general SOM includes (i) the amount of molecules associated with a node, (ii) vector of distances of these molecules to the node. Based on this information, the two main terms can be calculated:

1. Degree of similarity of molecular density distribution of sets A and B over a SOM. Expressed in $e^{-|p_{A,i}-p_{B,i}|}$, where $p_{A,i}$ is a density of molecules A in the node i, equal to $N_{moleculesAInNode}/N_{total}$, $p_{B,i}$ is the density of molecules B in a node correspondingly.

2. Degree of similarity in distances between the two sets in a node. This term as well reflects a chemical resemblance of molecules A and B since molecules of same chemical class/cluster would have analogous distances.
   Expressed in $e^{-(\left|\overline{d_{A,i}}-\overline{d_{B,i}}\right|/(d_{Amax,i}-d_{Amin,i}))}$, where $\overline{d_{A,i(B,i)}}$ is an

average distance of molecules A(B) to the node, $d_{Amax,i(Amin,i)}$ is a maximal (minimal) distance of molecules A to the node in this node. Range from 0 (molecules B are very different from molecules A) to 1 (molecules B are very similar to molecules A).

Thus,the formula for calculation the *affinity* of the projected data B to a SOM built on data A is:

$$S_a = \sum_{i=1}^{Nnodes} p_{B,i} * e^{-(|p_{A,i}-p_{B,i}|+\left|\overline{d_{A,i}}-\overline{d_{B,i}}\right|/(d_{Amax,i}-d_{Amin,i}))}$$

The score ranges from 0 to 1, where 0 means that the affinity of the projected data B to the SOM built on data A is very low. The maximal score could be achieved for the case when the density distribution of data B is as close as possible to the density distribution of data A and the average distances of molecules A and B per node are almost equal, meaning close chemical similarity of B to A in a node.

Table below shows the overall score for each SOM together with the contributions of each of the two terms.

	S_a	density[a]	distance[b]
SOM $QM9_{PC9projection}$	**0.8456**	0.9973	0.8480
SOM $PC9_{QM9projection}$	**0.9370**	0.9989	0.9381

[a]density term, $\sum_{i=1}^{Nnodes} p_{B,i}, i * e^{-(|p_{A,i}-p_{B,i}|}$

[b]distance term, $\sum_{i=1}^{Nnodes} p_{B,i} * e^{-(|\overline{d_{A,i}}-\overline{d_{B,i}}|/(d_{Amax,i}-d_{Amin,i}))}$

According to the result, the density term is very similar for both SOMs meaning that molecules B are distributed proportionally to molecules A over a SOM (Fig. 1, left). However, the distance term of QM9 SOM is lower compare to PC9 SOM. Fewer diversity of functional groups of QM9 leads to less universal SOM, upon which the PC9 molecules of uncommon classes would be projected mixed with the known classes. That will lead to lower chemical purity per a node and decrease the distance term (Fig. 1,right).

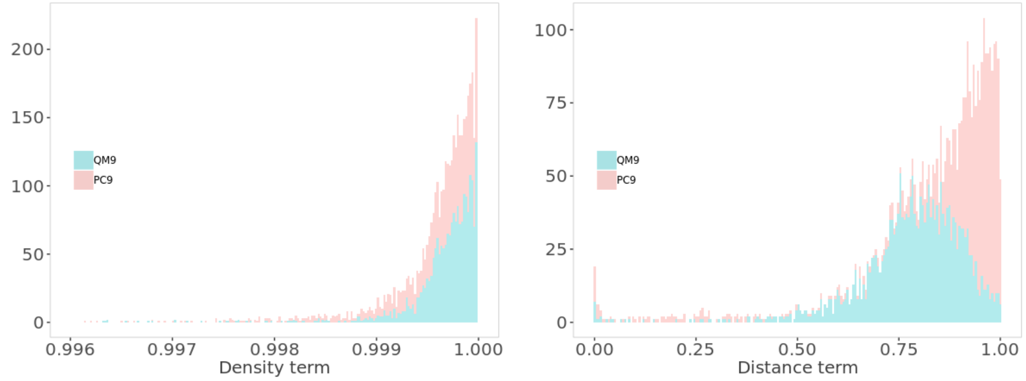

Figure 1: The density term $e^{-(|p_{A,i}-p_{B,i}|}$ and the distances term $e^{-(|\overline{d_{A,i}}-\overline{d_{B,i}}|/(d_{Amax,i}-d_{Amin,i}))}$ per node for SOM QM9 and SOM PC9

.

In summary, the formula accounts equally for similarity in density distribution and in chemical diversity and could be easily analyze by these terms. The contributions of these terms are weighted by the node's data density so that the most populated nodes would have bigger influence on the overall score. The formula is thus reflects the affinity and the comparability of the projected data to a given SOM.
